# Do Amplitudes of Water Level Fluctuations Affect the Growth and Community Structure of Submerged Macrophytes?

**DOI:** 10.1371/journal.pone.0146528

**Published:** 2016-01-06

**Authors:** Mo-Zhu Wang, Zheng-Yuan Liu, Fang-Li Luo, Guang-Chun Lei, Hong-Li Li

**Affiliations:** School of Nature Conservation, Beijing Forestry University, Beijing, China; Swiss Federal Research Institute WSL, SWITZERLAND

## Abstract

Submerged macrophytes are subjected to potential mechanical stresses associated with fluctuating water levels in natural conditions. However, few experimental studies have been conducted to further understand the effects of water level fluctuating amplitude on submerged macrophyte species and their assemblages or communities. We designed a controlled experiment to investigate the responses of three submerged macrophyte species (*Hydrilla verticillata*, *Ceratophyllum demersum* and *Elodea nuttallii*) and their combinations in communities to three amplitudes (static, ± 30 cm, ± 60 cm) of water level fluctuations. Results showed that water level fluctuating amplitude had little effects on the community performance and the three tested species responded differently. *H*. *verticillata* exhibited more growth in static water and it was negatively affected by either of the water level fluctuations amplitude, however, growth parameters of *H*. *verticillata* in two fluctuating water level treatments (i.e., ± 30 cm, ± 60 cm) were not significantly different. On the other hand, the growth of *C*. *demersum* was not significantly correlated with different amplitude treatments. However, it became more abundant when water levels fluctuated. *E*. *nuttallii* was inhibited by the two fluctuating water level treatments, and was less in growth parameters compared to the other species especially in water level fluctuating conditions. The inherent differences in the adaptive capabilities of the tested species indicate that *C*. *demersum* or other species with similar responses may be dominant species to restore submerged macrophyte communities with great fluctuating water levels. Otherwise, *H*. *verticillata*, *E*. *nuttallii* or other species with similar responses could be considered for constructing the community in static water conditions.

## Introduction

Submerged macrophytes play an important role in aquatic ecosystems [1–3] as they mediate some significant ecological processes such as purifying water, increasing nutrient retention and providing refuge or food for some organisms [3–5]. Submerged macrophyte communities are heavily influenced by both abiotic and biotic factors in their immediate environment like water level fluctuations [6,7], light intensity [2], nutrient availability [5,8], dreissenid invasion [9], and the algae cover [2].

Water level fluctuations, varying in both temporal and spatial scales in aquatic systems (i.e. lakes, streams and rivers), encompass not only the amplitude but also the frequency and regularity of change [6,10,11]. Fluctuating amplitude defined as the difference between maximum and minimum water levels mainly depends on regional conditions and anthropogenic factors [12,13]. For example, constructions of dams and land reclamations for agriculture use strongly alter the natural patterns of amplitude [11,14]. Amplitude of water level fluctuations may significantly change light availability, water pressure and other stability of factors in the aquatic environment [12,15]. For instance, water level fluctuations undergoing excessively high water level can sharply reduce the amount of light that reaches submerged macrophytes, while extremely low level may damage plants through wave action or desiccation [12]. Therefore, the variation of water level fluctuating amplitude caused by both natural and human factors may greatly influence the performance of submerged macrophyte species and their communities [10,16].

In some studies, moderate water level fluctuating amplitude was positively correlated with species-rich submerged macrophyte communities, while extreme fluctuation decreased the species richness [10]. However, other studies indicated that species richness of the submerged macrophyte community was reduced at small fluctuating amplitude only [17]. Such discrepancy may be attributed to different species that constitute the community exhibit varying responses to amplitudes of water level fluctuations particularly in their survival tactics [1,18]. In general, submerged macrophytes adapt to water level fluctuating amplitude through morphological plasticity and biomass reallocation [6,11,19]. Previous studies have revealed that moderate water level fluctuating amplitude can promote the distribution and growth of submerged macrophytes such as *Myriophyllum spicatum* [6], *Ottelia alismoides* [11], whereas extreme water level fluctuations inhibit their growth as they exceed the physiological limits of plant [12].

To our knowledge, previous studies mostly focused on the effects of water depth gradients, flooding rate, and other factors on the growth and performance of some submerged macrophytes [6,20–22]. To date, there is still very limited experimental evidence on how water level fluctuating amplitude affects the submerged macrophytes through comparisons of their responses at the species and community levels. In this study, we conducted a controlled experiment to explore the responses of three submerged macrophytes and their community structure to water level fluctuating amplitude. Specifically, we addressed the following questions: (1) how did the performance and structure of simulated and real submerged macrophyte communities respond to water level fluctuating amplitude? (2) how did the growth of each species respond to fluctuating amplitude when they are in monoculture or in mixture, respectively?

## Materials and Methods

### Plant materials

The study was conducted on three different submerged macrophytes, namely, *Hydrilla verticillata* (L.f.) Royle (Hydrocharitaceae), *Ceratophyllum demersum* L. (Ceratophyllaceae) and *Elodea nuttallii* (Planch.) H. St. John (Hydrocharitaceae). They are widespread in the aquatic habitat due to high capacity for vegetative propagation by producing clonal fragments and turions, and spread rapidly via clonal dispersals [2,6,23–26]. We chose these three species because the three submerged macrophytes can co-occur in relatively enclosed aquatic conditions like lakes, ponds, ditches [2,27–29], according to the literature and personal observation (by Yong-Yang Wang). To important, they are easily to obtain and commonly used for restoration of degraded aquatic ecosystems in China.

### Plant sampling and experimental design

In early-July 2013, we sampled stem fragments of the three species from lakes of the Winter Palace (40°00'15.96" N, 116°18'11.26" E) and the Olympic Green (40°00'43.50" N, 116°23'07.31" E) in Beijing, China. Total nitrogen and phosphorus in lake water of the Winter Palace were 0.280–0.980 mg L^-1^ and 0.019–0.044 mg L^-1^, respectively, in October 2008 [30]. The collection of plant materials was approved by the administrative department of Winter Palace and the Olympic Green in Beijing, China. The species we used in this study are not endangered or protected species. After cultivating for 10 days in the green house at Forestry Science Co., Ltd, of Beijing Forestry University in Beijing, we selected 135 shoot fragments of each species for the experiment. To minimize variances due to effects of size of initial shoot materials, each shoot was cut to approximately 10 cm long and had 8 nodes with side branches removed.

**Fig 1:**
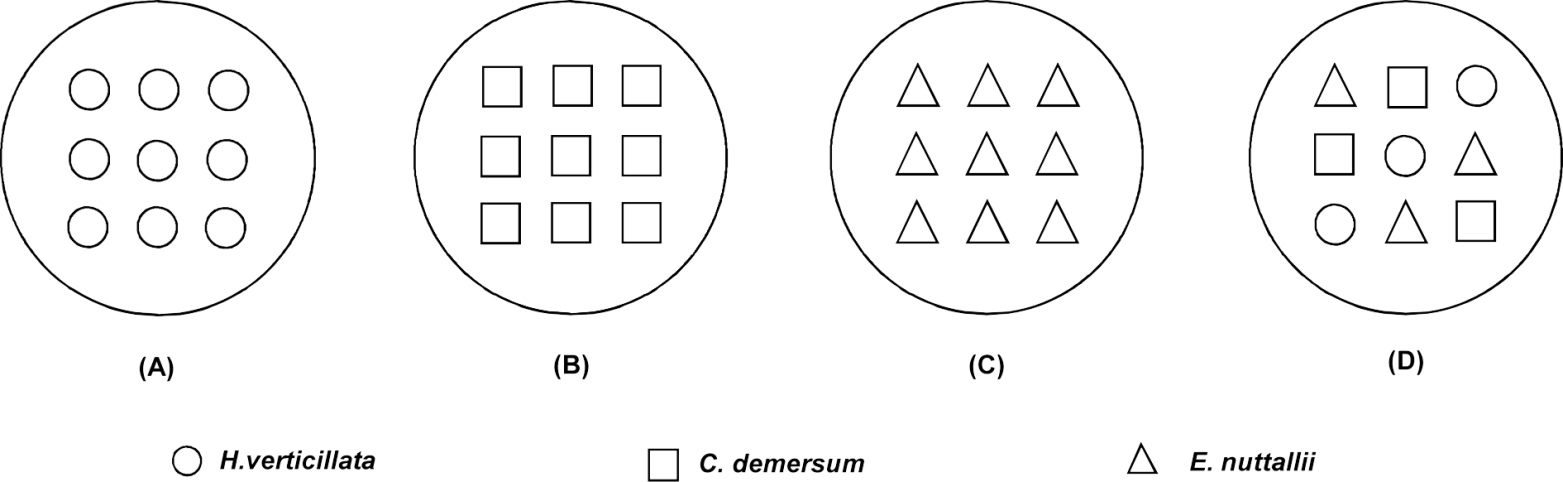
Cultivation methods. (A)-(C) monoculture. (D) mixture.

**Fig 2:**
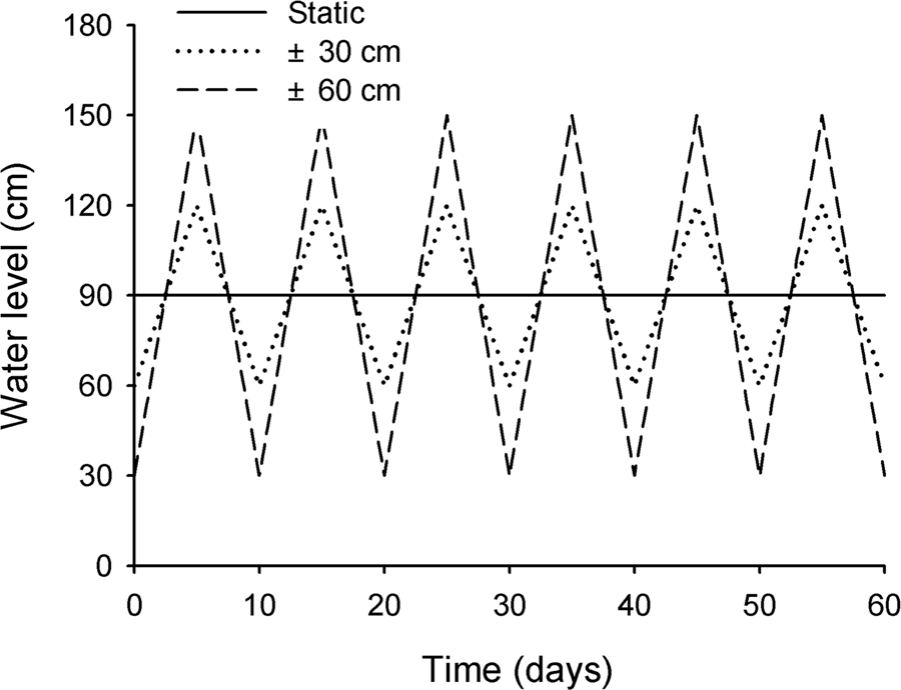
The amplitudes of water level fluctuation in this experiment.

The experiment took in a split-plot design with three fluctuating amplitudes (static, ± 30 cm, ± 60 cm) as the main plot factor, two cultivation methods (*H*. *verticillata*, *C*. *demersum* and *E*. *nuttallii* in both monoculture and mixture) as the sub-plot factor. Each treatment had 5 replicates (five independent plastic tanks each containing one replicate for each of the 12 treatments). On July 14, the shoot fragments were planted in plastic pots (14 cm in bottom diameter, 21 cm in top diameter and 23 cm in height) filled with a 18-cm-deep mixture of river sand and soil at 1:1 volume ratio. The substrate was further covered by a 2-cm-deep layer of quartz sand to reduce turbidity. In monoculture treatments, nine shoot fragments of each species were randomly planted in three rows and three columns per pot. In mixture pot consisting of the three species, three fragments of the same species were planted in different rows and columns arranged following a Latin square approach (Fig 1). Each of the pot was placed into a larger mesh container separated by mesh from each other to avoid plants growing into the adjacent pot. The 12 mesh containers belonging to the same replicate were then suspended in one of the five plastic tanks (150 cm in diameter × 170 cm in depth) full of tap water (TN: 0.670 ± 0.050 mg L^-1^, TP: 0.044 ± 0.011 mg L^-1^, TK: 1.967 ± 0.018 mg L^-1^, pH: 8.170 ± 0.036; mean ± SE, n = 3). All pots were placed at initial water level of 90 cm for 10 days to acclimatize. Then water level fluctuated around the initial water level of 90 cm in all treatments. Three amplitudes of water level fluctuations were applied: static, ± 30 cm, ± 60 cm (Fig 2). In other words, the fragments were exposed to three fluctuating amplitude treatments (static, ± 30 cm, ± 60 cm) by vertically adjusting the pots through the ropes. The fluctuations were performed around initial water level (90 cm) with the start of lower limit of the amplitudes (Fig 2). Therefore, the corresponding flooding of draw-down rates were 0, 12, and 24 cm per day with 10 days as a cycle. The experiment lasted 60 days from July 15 to September 14, 2013. Light intensities at water surface and in the water column (about 30 cm water depth) on sunny day were 1352.400 ± 173.300 and 589.300 ± 39.600 μmol m^-2^ s^-1^ (mean ± SE, n = 3), respectively, measured by a Li-COR UWQ-4341 sensor at noon on 25 July.

Our experiment was carried out in the open air. In order to reduce the effects of high outdoor temperature and strong sunlight on plant growth, each tank was surrounded by shading mat and covered with sunshading net. During the experiment, tap water of each tank was replaced every 15 days to maintain water quality and replenished every 3–4 days. Moreover, impurities such as algae were cleaned from tanks using a small, circular fishnet (9 cm in diameter and 0.5 mm in mesh size) when they were clearly visible in the water body.

### Harvest and measurements

At the end of the experiment (September 15), all surviving submerged macrophytes were carefully harvested and sorted by species. Growth parameters such as biomass, stem length and node number of each plant in monoculture and mixture were then determined. However, it was difficult to measure stem length and node number for all plants as the shoots were delicate and tended to be damaged and broken into numerous fragments during harvest. Therefore, five stem fragments of each species in each pot were randomly selected as representative samples from which growth parameters were measured. In addition, the remaining parts of each species in each pot were also weighed. For biomass measurements, the plants were oven dried at 70°C for 48h and weighed.

### Data analysis

Stem length and node number per unit biomass of each species in each pot were firstly determined according to the data of the 5 representative fragments. Then species stem length in each pot was derived by multiplying species biomass (samples and remaining part biomass) with stem length per unit biomass. Similarly, we derived node number for each species in each pot.

At the community level, there were two types of communities. The simulated communities (Code as SC) were projected communities without the interspecific interaction among the three species, while the real communities (Code as RC) were actual communities with the interspecific interaction among the three species (Fig 1D). The simulated communities were constructed basing on the monoculture data of each amplitude treatment belonging to the same replicate. Growth parameters of simulated communities (Code as SC_G_) were determined using the following formula: ${SC}_{G}=\frac{1}{n}\sum Gi$ (*i* = 1,2…n), where n is the number of the macrophyte species (n = 3) and G*i* is the growth parameter (biomass, stem length, node number) of species *i* [1]. Moreover, growth parameters of the real communities (Code as RC) were defined as the sum of the corresponding variables of all the three component species in a mixture pot. Furthermore, the proportion of each species in the SC and RC based on biomass, stem length and node number were also calculated as measures of relative abundance.

At species level, we calculated biomass, stem length and node number per individual shoot of each species both in monoculture and in mixture. In monoculture, the species growth parameters of each pot were divided by nine (i.e., the shoot number in a monoculture pot), while in mixture, the growth parameters of each species in each pot were divided by three (i.e., the shoot number of each species in a mixture pot).

Split-plot ANOVA was applied to determine the effects of fluctuating amplitude, community type (at community level) or cultivation method (at species level), and their interaction on overall growth parameters (biomass, stem length, and node number) of the communities and individual species. Then, one-way ANOVA was performed to test the effects of water level fluctuating amplitude on performance and species composition of the SC and RC, and each species performance in monoculture and in mixture, respectively. If a significant treatment effect was detected, post hoc pairwise comparisons were conducted to examine the differences between amplitude treatments using Duncan multiple comparisons. For all community and species data, Levene’s test was carried out before variance analysis and data that did not fit the assumption of ANOVA were logarithmically transformed [log_10_(x)] to improve normality and homoscedasticity. All analyses were conducted in SPSS 19.0 software (SPSS, Chicago, IL, USA).

## Results

### Community performance and species composition

**Fig 3:**
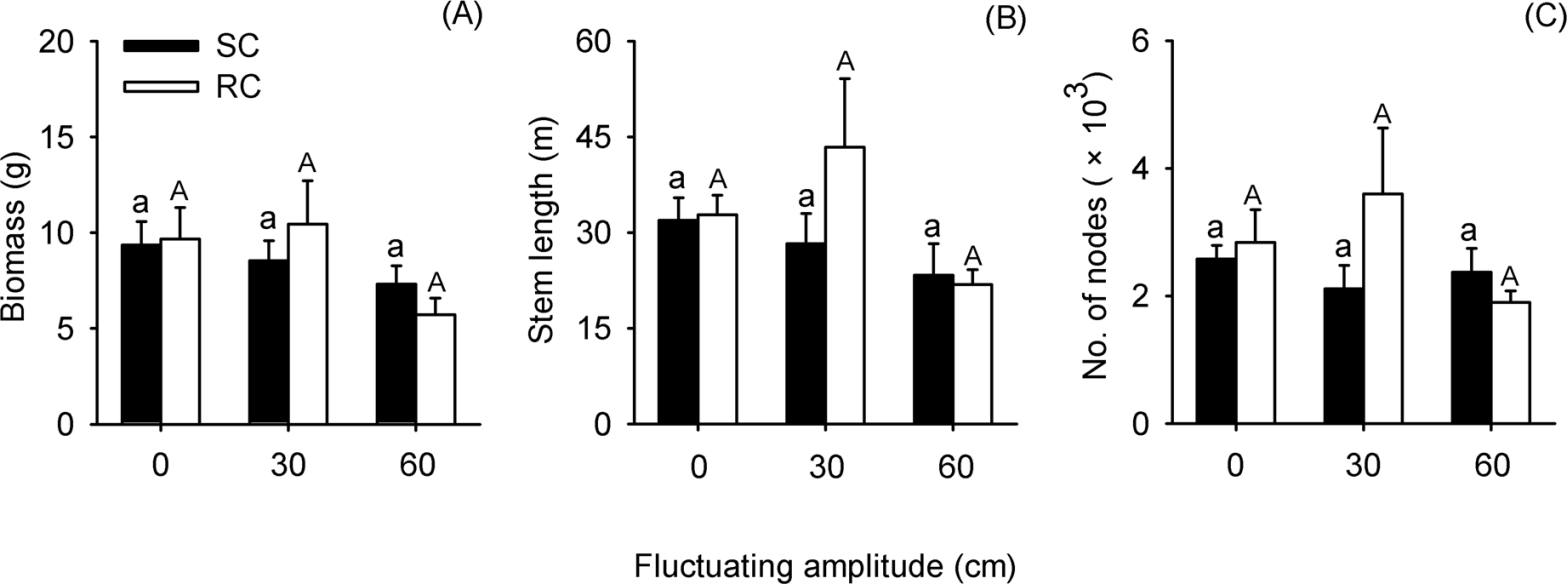
Effects of water level fluctuating amplitudes on performance of simulated community (SC) and real community (RC). Mean values (± SE) of biomass (A), stem length (B), and number of nodes (C) are given. For different community types, bars sharing the same letters are not significantly different at *P* = 0.05.

Fluctuating amplitude only had significant effects on stem length of the submerged macrophyte communities (Table 1). However, the growth parameters of the two types of communities did not vary with different water level fluctuating amplitude treatments (Fig 3). Furthermore, community type and the interaction between the two factors had insignificant effect on the performance of the submerged macrophyte communities (Table 1).

**Table 1:** Split-plot ANOVA results for effects of water level fluctuating amplitude and community type on biomass, stem length and node number of submerged macrophyte communities.

	Water level fluctuating amplitude (A)		Community type (T)		A×T	
parameters	*F*_2,24_	*P*	*F*_1,24_	*P*	*F*_2,24_	*P*
Biomass	1.951	0.204	0.048	0.830	1.251	0.321
Stem length ^a^	5.114	0.037	0.976	0.343	0.897	0.433
Node number ^a^	1.597	0.261	0.532	0.480	1.862	0.198

^a^ log_10_(x)-transformed.

**Fig 4:**
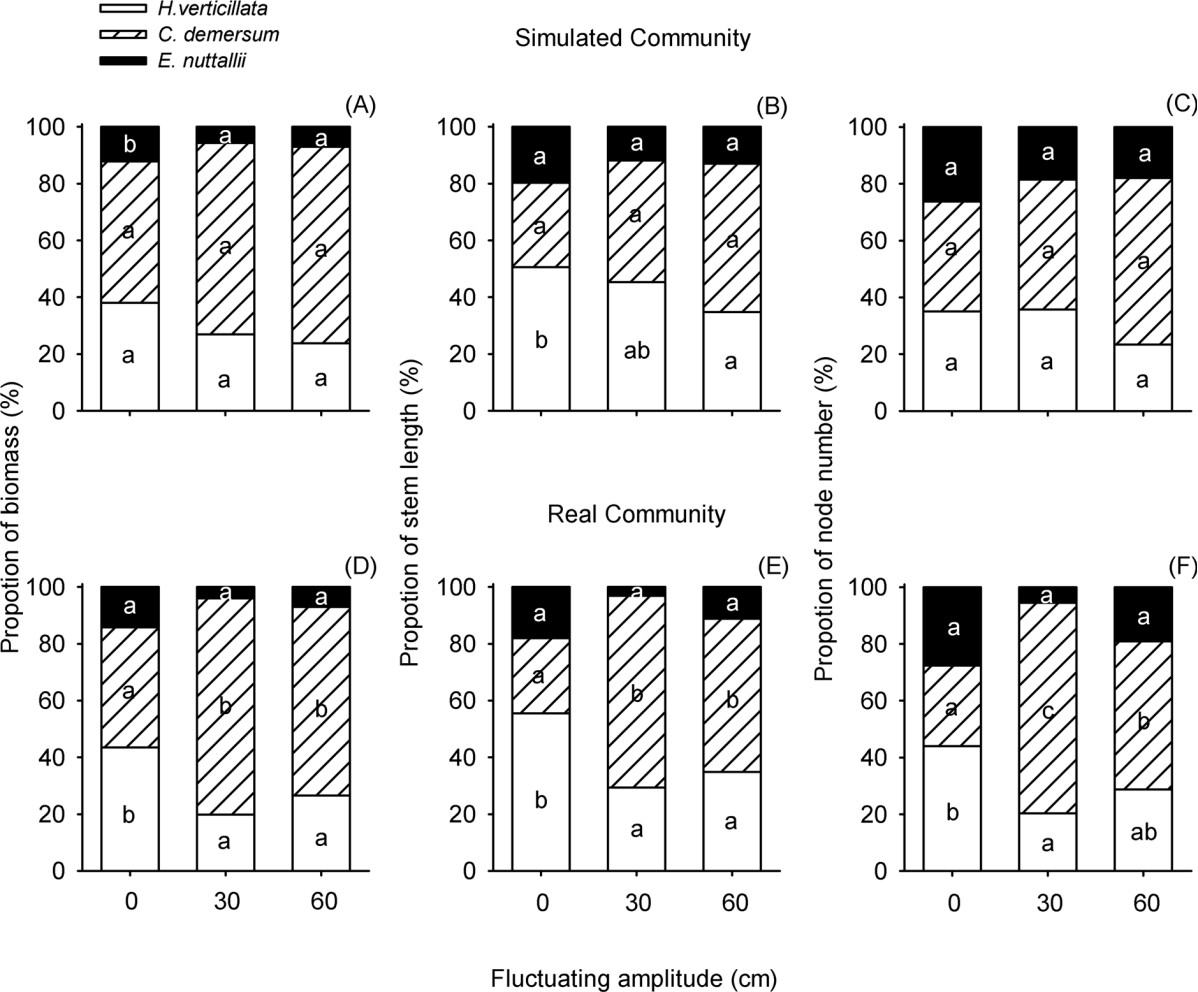
Effects of water level fluctuating amplitudes on species composition of simulated community (SC) and real community (RC). The proportions of biomass (A, D), stem length (B, E) and node number (C, F) of three submerged macrophytes are given. For each species, different letters show significant differences among treatments at *P* = 0.05.

For the simulated community, biomass proportion of *E*. *nuttallii* was significantly larger in the 90 cm-static water than in the fluctuating water. However, no significant difference was found between the ± 30 cm and ± 60 cm water level fluctuating amplitude treatments (Fig 4A). Stem length proportion of *H*. *verticillata* was also higher in 90 cm-static water, and smallest in the ± 60 cm water level fluctuating amplitude treatment (Fig 4B). For the real community, the proportions of biomass, stem length and node number of *H*. *verticillata* were significantly higher in the 90 cm-static treatment than in the other two water level fluctuating amplitude treatments (Fig 4D–4F). In contrast, *C*. *demersum* demonstrated greater proportion in the amplitudes of ± 30 cm or ± 60 cm compared with the control treatment of 90 cm-static water level (Fig 4D–4F). The proportion of *E*. *nuttallii*, which was lowest in the community, showed no apparent response to water level fluctuating amplitude (Fig 4D–4F).

### Growth of individual species

**Fig 5:**
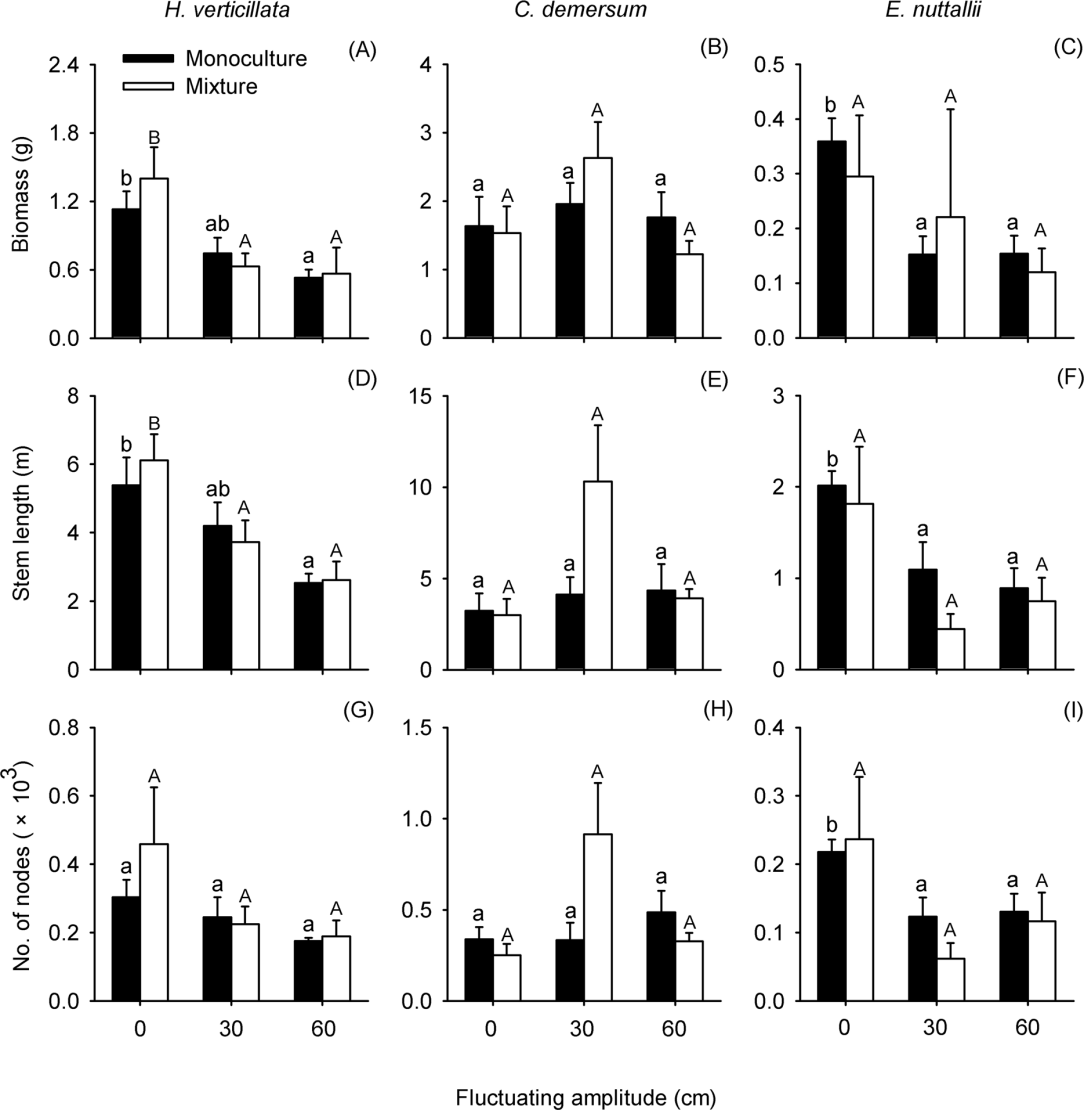
Effects of water level fluctuating amplitudes on growth of individual species. Mean values (± SE) of biomass (A-C), stem length (D-F) and number of nodes (G-I) are given. For different cultivation methods, bars sharing the same letters are not significantly different at *P* = 0.05.

The biomass and stem length of individual shoot of *H*. *verticillata* showed significant responses to water level fluctuating amplitude (Table 2). In both cultivation methods, biomass (1.131 ± 0.155 g in monoculture; 1.401 ± 0.275 g in mixture) and stem length (5.388±0.809 m in monoculture; 6.118±0.764 m in mixture) of *H*. *verticillata* in the 90 cm-static water were markedly higher than those in the other treatments and the two parameters reduced at higher amplitudes. The same pattern was true for the node number although there was no significant difference between static and fluctuating water (Fig 5A, 5D and 5G). For *C*. *demersum*, no growth parameter seemed to be affected by water level fluctuating amplitude (Table 2), regardless of cultivation method (Fig 5B, 5E and 5H). In comparison, all measured growth parameters of *E*. *nuttallii* were significantly influenced by water level fluctuating amplitude (Table 2), but patterns were not consistent in the two different cultivation methods. In monoculture, growth parameters were significantly higher at 90 cm-static water level, while in mixture this difference was less pronounced (Fig 5C, 5F and 5I). In addition, cultivation method affected the stem length of *E*. *nuttallii*, while the interaction between the two factors had significant effect on the node number of *C*. *demersum* (Table 2).

**Table 2:** Split-plot ANOVA results for effects of water level fluctuating amplitude and cultivation method on biomass, stem length and number of nodes of each individual shoot of submerged macrophytes.

	Water level fluctuating amplitude (A)		Cultivation method (M)		A×M	
Parameters	*F*_2,24_	*P*	*F*_1,24_	*P*	*F*_2,24_	*P*
***H*.*verticillata***						
Biomass	9.741	0.007	0.254	0.624	0.780	0.480
Stem length	19.395	0.001	0.079	0.783	0.762	0.488
Node number ^a^	4.203	0.057	0.063	0.805	0.510	0.613
***C*. *demersum***						
Biomass	2.434	0.149	0.003	0.959	2.475	0.126
Stem length ^a^	3.866	0.067	0.613	0.449	2.184	0.155
Node number ^a^	2.170	0.177	0.001	0.975	5.116	0.025
***E*. *nuttallii***						
Biomass ^a^	7.096	0.017	4.037	0.068	0.336	0.721
Stem length ^a^	7.253	0.016	5.089	0.044	0.300	0.746
Node number ^a^	5.907	0.027	4.172	0.064	0.278	0.762

^a^ log_10_(x)-transformed.

## Discussion

### Community performance and species composition

Our results suggested that water level fluctuating amplitude had little effects on the community performance. Previous studies indicated that the change in performance of the community is mainly driven by responses of species, especially abundant species, in the community [1]. In this study, the three species constituting the two types of submerged macrophyte communities responded differently to fluctuating amplitude. The growth and proportion of *H*. *verticillata* and *E*. *nuttallii* tended to be smaller in the fluctuating water (± 30 cm, ± 60 cm) than in the static water, whereas *C*. *demersum* exhibited the opposite patterns. Because of such species-specific and contradictory responses of the three species (Figs 4 and 5), the differences of community performance among different fluctuating amplitudes were counteracted (Fig 3).

The species composition difference between simulated and real communities under different fluctuating amplitudes was rarely detected in other studies. In this study, obvious differences in the proportion of each species between the two types of communities appeared in ± 30 cm amplitude treatment (Fig 4). It likely resulted from the difference of interspecific interaction among three species in different fluctuating amplitudes. Interspecies interaction is closely associated with the hydrological environment and suitable water level can even intensify the interspecies competition [31]. In our study, the amplitude of ± 30 cm could be the moderate amplitude for the tested species in which competition among them was intensified.

### Growth of individual species

Water level fluctuating amplitude can cause physical, chemical and biological changes in a water body, which in turn influence the growth of submerged macrophytes. For example, hydraulic forces generated by fluctuating water often bring mechanical stress that induces stem breakage, uprooting or other mechanical damages to the submerged macrophytes [32–34]. Additionally, water level fluctuations can lead to the changes of water depth, which is usually associated with light availability, a driving force for the growth of plants [21,35]. Such effects may trigger adaptive traits of submerged macrophytes for responding to resource limitations and stresses caused by constantly changing environment.

The three submerged macrophyte species responded differently to fluctuating amplitude. *H*. *verticillata* was the most abundant species in real communities when water level was static. Similarly, Zhang *et al*. [1] found that *H*. *verticillata* could easily become the superior competitor in the submerged macrophyte communities in undisturbed conditions. *H*. *verticillata* has high polymorphic plasticity to water level fluctuation, which is associated with its low lignin content [6,21]. However, lack of lignin leads to more fragile stems that are easily breakable in wave and mechanical disturbance [6]. Furthermore, the cyclic water level fluctuations may result in intermittent periods of resource limitation for *H*. *verticillata* and the degree of limitation should be commensurate with the amplitude of water level fluctuations [36,37]. Consequently, this species was adequate in static water and responded negatively to increasing fluctuating amplitudes. *H*. *verticillata* can adapt and grow rapidly by developing longer stems and accumulating more biomass in slow or static flowing water due to its low light requirement and efficient use of nutrient [1,23,38]. Therefore, *H*. *verticillata* eliminated other species and dominated in the community through interspecific competition in static water conditions [23,39].

In this study, *C*. *demersum* was insensitive to amplitudes of water level fluctuations. It might be due to the fact that *C*. *demersum* needs longer time to exhibit phenotypic adaptations to changing water level [11]. In fluctuating treatments, the draw-down rates of water level were 12 and 24 cm per day, and the fluctuating frequency might be high for this species, resulting in delayed or unapparent plastic response. Moreover, *C*. *demersum* often inhabit deeper waters due to its low light compensation point [40]. Therefore, light limitation generated by water level fluctuation seems to be not the primary factor in determining the growth responses of *C*. *demersum*. In fact, when water fluctuated (± 30 cm, ± 60 cm), *C*. *demersum* dominated the submerged macrophyte community. Zhu *et al*. [32] also found that *C*. *demersum* was most adaptive to floods due to its stronger and more flexible stems that are well adapted to fluctuating water.

When planted alone, *E*. *nuttallii* seemed to be inhibited by fluctuating water. This finding is similar to previous reports on the responses of *E*. *nuttallii* to water turbulence [34,41]. *E*. *nuttallii* would shorten stem length and expand radially to adapt to hydrodynamic forces [34], while the loss of biomass is closely related to leaf senescence [41] due to suppressed chlorophyll production and decreasing photosynthesis by water level turbulence [34]. However, when planted with other species, the significant differences of growth parameters among different amplitude treatments were eliminated, which might be caused by the influence of interspecies competition. Generally, species with the same level of need or requirement for resources such as light, nutrients and other resources strongly compete with each other when they grow up together [42]. In this study, the growth of *E*. *nuttallii* seemed to be negatively affected by interspecific competition when they were planted with other species. Similar finding was reported in the growth rate of *E*. *nuttallii*, which was inhibited by *Salvinia natans* due to the competition for resources [42]. Furthermore, *H*. *verticillata* is the stronger competitor compared to *E*. *nuttallii* in the short-term static water, since the upper canopy formed by *H*. *verticillata* shades and inhibits the development of *E*. *nuttallii* [43]. On the other hand, the decreased growth of *E*. *nuttallii* was likely induced by its weaker competitiveness relative to *C*. *demersum* in water fluctuation conditions.

According to the current results, it could be suggested that water level fluctuating amplitudes have little effects on the performance of the submerged macrophyte community due to species-specific responses of different submerged macrophytes. In addition, moderate amplitude can intensify interspecies competition in the submerged macrophyte community. In application, *C*. *demersum* or other species with similar responses could be ideal for restoration of submerged macrophyte communities in fluctuating water. In contrast, to restore submerged macrophyte communities in conditions with static water, *H*. *verticillata* and *E*. *nuttallii* or other species with similar responses could be considered for reconstructing the community. Furthermore, other environmental factors such as nutrient, light should also be considered besides the amplitude of water level fluctuation [19,42]. Therefore, more attention should be paid to the influence of fluctuating amplitude on submerged macrophytes in different environmental conditions in the further studies.

## Supporting Information

S1 Table Data used from the experiment in analyses. (XLSX)
